# High-flow vs conventional oxygen therapies for acute cardiogenic pulmonary edema following hip fractures and surgery in elderly patients

**DOI:** 10.3389/fonc.2025.1520687

**Published:** 2025-04-30

**Authors:** Caizhe Ci, Xiao Tong, Weiyan Tai, Xiaoyong Geng, Yu Han, Xiaojun Zhang

**Affiliations:** ^1^ Department of Cardiovascular Medicine, Third Hospital of Hebei Medical University, Shijiazhuang, Hebei, China; ^2^ Department of Joint Surgery, First Hospital of Hebei Medical University, Shijiazhuang, Hebei, China

**Keywords:** hip fracture, acute cardiogenic pulmonary edema, high-flow oxygen therapy, conventional oxygen therapy, oxygen saturation. level of evidence: therapeutic study, level Ia

## Abstract

**Purpose:**

This prospective, randomized controlled study aimed to compare the effects of high-flow oxygen therapy and conventional oxygen therapy in the treatment of acute cardiogenic pulmonary edema following hip fractures and surgery in elderly patients.

**Methods:**

From February 2018 to October 2023, 124 patients diagnosed with acute cardiogenic pulmonary edema following hip fractures and surgery were randomly assigned to the high-flow oxygen therapy group (n=62) or conventional oxygen therapy group (n=65). Partial pressure of oxygen (PO_2_) and blood oxygen saturation (SPO_2_) were assessed 60 minutes after the treatments. A P value <0.05 was considered statistically significant.

**Results:**

There were significant differences in PO_2_ (66.2 ± 3.3 mmHg *vs* 62.1 ± 3.4 mmHg, P<0.05) and SPO_2_ (97.8 ± 2.1 mmHg *vs* 94.2 ± 1.7 mmHg, P<0.05) between the groups.

**Conclusion:**

In the treatment of elderly patients with ACPE following hip fractures and surgery, high-flow oxygen therapy may be performed to improve ventilation when acute cardiogenic pulmonary edema does not significantly improve within 15 minutes of conventional oxygen therapy.

## Introduction

The incidences of hip fractures are 19% in men and 25% in women. Owing to trauma and a bedridden state, 14% of non-operative and perioperative patients suffer from acute heart failure, resulting in acute cardiogenic pulmonary edema (ACPE) with a mortality rate of 15% (1–4). Treatment of ACPE following hip fractures and surgery has exceptional clinical challenges (5). Currently, supplemental oxygen therapy is a vital component, but the optimal oxygen delivery method is still controversial (3).

ACPE represents a common and serious non-operative and perioperative condition in hip fracture patients (6, 7). ACPE is caused by acute decompensated heart failure and pulmonary edema in the absence of heart disease (primary fluid overload due to blood transfusion), trauma, severe hypertension, renal artery stenosis, and severe renal disease (8). Currently, there is no gold standard for diagnosing ACPE. However, the generally accepted diagnostic criteria include a history of trauma, organic heart disease (ischemia with or without myocardial infarction, exacerbation of chronic systolic or diastolic heart failure, or aortic valve dysfunction.), sudden or worsening dyspnea, coughing up white or pink phlegm with bubbles, peripheral cyanosis, an increased heart rate (>126 beats/min), spontaneous breathing (>25 breaths/minute), and a bedridden state, etc. (9) The laboratory tests (slight elevation of cardiac troponin levels), echocardiography (normal or increased left ventricular systolic function), and chest X-rays (pulmonary edema) are also helpful. On chest X-rays, most diseases show a bilateral, symmetrical opacity occupying the central zones of the lungs, which may develop a classic “butterfly shadow” (10).

In conventional oxygen therapy (COT), oxygen is applied through low-flow (< 5 L/min) or moderate-flow (5–15 L/min) delivery devices or a combination thereof (nasal prong, mask with or without oxygen reservoir, and Venturi mask systems) (11). The drawbacks include a lack of precision of exact oxygen delivery, insufficient heating and humidifying, and poor tolerance (12). High-flow oxygen therapy (HFOT) was first patented by the Transpirator Technologies, Inc. in 1988 (13). It is commonly performed in critical care settings and high-dependency care units due to non-invasive and easy-to-use therapy (14). Currently, HFOT has become more popular because it delivers a warm, humidified flow (> 60 L/minute) through a soft nasal cannula (15). Many studies have shown the beneficial effect of COT *versus* HFOT on ACPE in blood gas analysis, heart rate, and respiratory rate (16–19). Nevertheless, those outcomes are based on adult general ward patients. Currently, whether those results are comparable in elderly patients with hip fractures and surgery is still not reported in the literature.

This prospective, randomized controlled study aimed to compare the effects of COT and HFOT in the treatment of elderly patients with ACPE following hip fractures and surgery.

## Materials and methods

The institutional review boards of the Third Hospital of Hebei Medical University approved the study (W2020-011-1). Informed consent was obtained from each patient. Clinical trial has been registered (e.g., clinicaltrials.gov) and registration number is pending.

From February 2018 to October 2023, patients with a diagnosis of ACPE following hip fractures and surgery were admitted and treated in our hospital. The diagnosis of ACPE was established based on the diagnostic criteria, as shown above (9, 10). The inclusion criteria for the study were (1) adult patients aged ≥ 55 years; (2) a confirmed diagnosis of hip fractures and ACPE (4, 5); (3) unilateral or bilateral hip involvement; (4) applying conventional oxygen therapy for 10 minutes to achieve a mask inhaled oxygen saturation (FiO_2_, fraction of inspired oxygen) of 50% and to increase blood oxygen saturation (SPO_2_) from 90% to 96% (or SPO_2_/FiO_2_ from 176 to 192); and (4) respiratory rates between 28 and 35 breaths/minute. The exclusion criteria included (1) age <55 years; (2) ACPE secondary to diseases, injuries other than hip fractures or concurrent injuries; (3) hip fracture treated non-operatively or operatively; (4) acute respiratory distress syndrome, sepsis, pneumonia, alveolar hemorrhage, or neoplasia; (5) noncardiogenic pulmonary edema due to septic shock or acute respiratory distress syndrome; (6) recurrent acute heart failure within 1 month; (7) cardiogenic shock; (8) concomitant chronic renal failure and liver failure; (6) acute exacerbation of chronic obstructive pulmonary disease; (9) type II respiratory failure; (10) development of worse hemodynamics within 15 minutes (SPO_2_ < 85%, respiratory rate > 40 breaths/minute, consciousness disorder, and intolerance of COT); (11) loss of follow-up or died; and (12) declined to participate the study. Moreover, if a patient had multiple hospital admissions due to ACPE, only the first admission was included.

Based on the types of oxygen therapies, we randomly and blindly assigned the eligible patients to the COT group and HFOT group using a computational pseudorandom number generator.

### COT

The patient lied in the semi-recumbent or sitting position. The patient was applied morphine sedation, diuretics, inotropic drugs, vasoactive drugs, and other treatments. The patients were given 24% oxygen *via* nasal prongs at flow rates of 2–3 L/minute or 28% oxygen *via* a Venturi mask at flow rate of 4 L/minute or a nasal cannula at flow rates of 1–2 L/minute. The goals were to achieve FiO_2_ of 50% and maintain SPO_2_ values between 90% and 96%.

### HFOT

The patient was placed in the same position, and similar medications were applied. The patient was treated with an Airvo 2 system (Fisher & Paykel Healthcare Optiflow, Auckland, New Zealand) through a nasal cannula or tracheostomy interface. We initially set the oxygen concentration to 40% with gases heated up to 37°C and 100% relative humidity. We set the mixed air-and-oxygen flow rate at 60 L/minute to keep the SPO_2_ value > 90%. Every 5 minutes, we increased or reduced oxygen saturation by 10% based on SPO_2_ values. The maximal oxygen concentration was 50% even if the SPO_2_ value > 90%.

### Outcome evaluation

We compared the two oxygen therapies in partial pressure of oxygen (PO_2_) and SPO_2_ measured before the treatments and 60 minutes after treatment starts. We determined the proportion of oxygenated hemoglobin in arterial blood with spectrophotometry. We recorded continuous non-invasive blood pressure. We obtained the laboratory tests before and during the treatments. We assessed heart failure based on the New York Heart Association Functional Classification (20), which allocated the patients to 4 categories based on the limitations of physical activity (I, no limitation; II, slight limitation; III, marked limitation; and IV, symptoms of heart failure at rest). We assessed the level of consciousness using the Glasgow Coma Scale (21) based on the patient’s ability to perform eye movements, speaking, and body movements (0, severe abnormal; 6, normal). We used the Borg’s modified scale (22) to evaluate the level of shortness of breathing (between 0 and 10). Patient comfort was assessed using the visual analog scale (0, very uncomfortable; 10, very comfortable) (17). We used the 5-point Likert scale (23) (very satisfied, somewhat satisfied, neither satisfied nor dissatisfied, somewhat dissatisfied, and very dissatisfied) to assess nurses’ attitude of skill difficulty, requirements, load intensity, operating risks, and willingness based on retrospective questioning within 24 hours.

### Statistical analysis

Quantitative variables were described as mean and standard deviation for symmetric distribution or median and interquartile range for asymmetric distribution. We used the Mann-Whitney U-test and *t-*test to determine whether there were any significant differences between the groups. We used the chi-square test to examine the association between categorical variables and frequencies. A 95% confidence interval was used to estimate the range of the true value. Differences were considered statistically significant at P<0.05. The collected data were analyzed with the Statistical Package for Social Sciences 24.0 (SPSS, Inc., Chicago, Ill).

## Results

**Figure 1:**
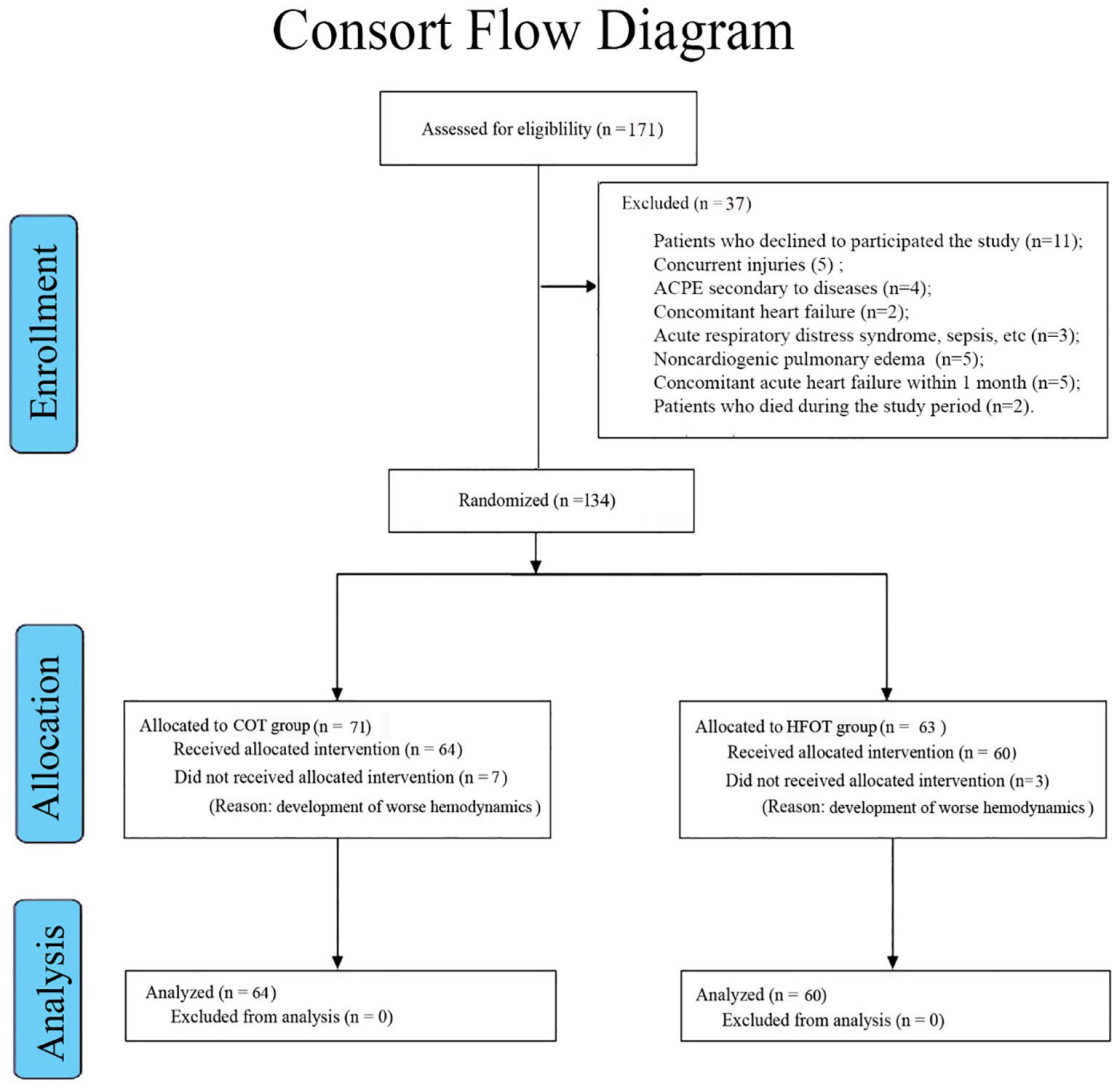
A flowchart showing the selection and allocation for 124 patients. COT, conventional oxygen therapy. HFOT, high-flow oxygen therapy.

**Figure 2:**
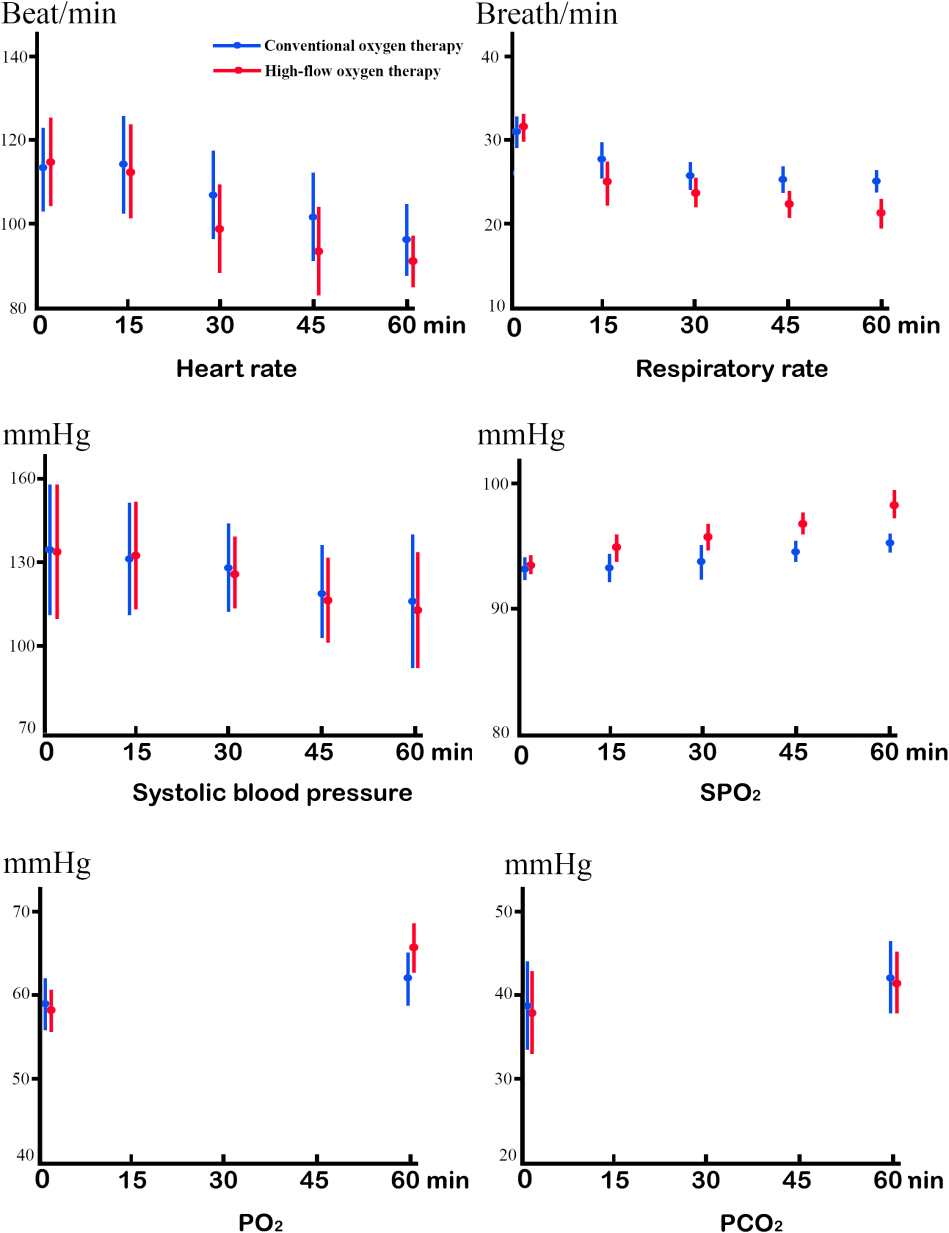
Linear mixed model of the mean changes for the COT group *versus* HFOT group, in heart rate, respiratory rate, systolic blood pressure, oxygen saturation (SpO_2_), partial pressure of oxygen (PO_2_), and pressure of carbon dioxide (PCO_2_).

From among 171 potential patients, we excluded 37 patients involving the patients who declined to participated the study (n=11); concurrent injuries (n=5); ACPE secondary to diseases (n=4); concomitant heart failure (n=2); acute respiratory distress syndrome, sepsis, pneumonia, alveolar hemorrhage, or neoplasia (n=3); noncardiogenic pulmonary edema due to septic shock or acute respiratory distress syndrome (n=5); concomitant acute heart failure within 1 month (n=5); and patients who died during the study period (n=2) (**Table 1**). We excluded 10 patients due to the development of worse hemodynamics. No patient lost to follow-up for 1 year. A total of 124 patients were finally analyzed (**Figure 1**). The age of the COT group (n=64) was 69.22 ± 10.07 years (range, 57–98 years). There were 42 male patients and 22 female patients. There were femoral neck fractures (n=56), greater trochanteric fractures (n=5), acetabular fractures (n=5), and femoral head fractures (n=2). Surgery was performed in 42 patients. The age of the HFOT group (n=60) was 68.55 ± 11.33 years (range, 55–89 years). There were 44 male patients and 16 female patients. There were femoral neck fractures (n=53), greater trochanteric fractures (n=3), acetabular fractures (n=7), and femoral head fractures (n=4). Surgery was performed in 47 patients. Patients’ concomitant diseases included diabetes, hypertension, coronary heart disease, acute coronary syndrome, dyslipidemia, cardiomyopathy, etc. There were no significant differences in age, sex, fracture site, or concomitant diseases (**Table 1**, **Figure 2**).

**Table 1:** Demographic and clinical characteristics for 124 patients.

	COT group	HFOT group		
	(n=64)	(n=60)	*t*	P value
Age (mean, range, year)	69.22 ± 10.07 (57-98)	68.55 ± 11.33 (55-89)	1.57	0.121
Sex (male: female)	42: 22	44: 16	0.05	0.705
Smoking (n)	13	16	0.4	0.758
Alcohol (n)	8	6	0.3	0.205
Cause (n)				
Road traffic accident	11	9	1.14	0.252
Fall	50	48		
Sports	2	3		
Work	1	0		
Time from injury to admission (day)	1.58 ± 3.33(0-6)	1.76 ± 2.07 (0-5)	1.255	0.258
Fracture site (n)				
Femoral neck fractures	56	53	0.403	0.714
Greater trochanteric fractures	5	3		
Acetabular Fractures	5	7		
Femoral head fracture	2	4		
Injured side (n)				
Left	38	28	0.308	0.787
Right	22	25		
Both	4	7		
Operative: nonoperative (n)	42: 22	47: 13	0.286	0.823
ACPE onset (n)				
Preoperative	11	10	-0.945	0.444
Intraoperative	1	2		
Postoperative	30	35		
Implant (n)				
Nail	7	3	-0.608	0.586
Plate	2	1		
Screw	27	31		
Arthroplasty	12	19		
Concomitant disease (n)				
Diabetes	22	15	-0.27	0.794
Hypertension	12	23		
Coronary heart disease	18	20		
Acute coronary syndrome	8	10		
Dyslipidemia	11	8		
Cardiomyopathy	14	18		
Atrial fibrillation	14	20		
Cerebrovascular disease	24	16		
Previous acute heart failure	16	14		

ACPE, acute cardiogenic pulmonary edema; COT, conventional oxygen therapy; HFOT, high-flow oxygen therapy.

We found no significant differences between the groups in pre-treatment heart rate, respiratory rate, systolic blood pressure, SPO_2_, pH, PO_2_, PCO_2_, HCO_3_, or lactic acid (**Table 2**, **Figure 2**). We found significant differences in heart rate (113.5 ± 11.8 *vs* 111.6 ± 10.7, P<0.001) 15 minutes after treatment starts, respiratory rate (25.4 ± 1.6 *vs* 23.1 ± 1.8, P<0.001) 30 minutes after treatment starts, SPO_2_ (93.7 ± 2.1 *vs* 95.3 ± 1.9, P<0.001) 15 minutes after treatment starts, and PO_2_ (62.1 ± 3.4 vs 66.2 ± 3.3, P<0.001) 60 minutes after treatment starts. We found significant differences in heart rate between 15 minutes and 30 minutes after treatment starts of the COT group (113.5 ± 11.8 *vs* 105.3 ± 11.1, P<0.001) and HFOT group (111.6 ± 10.7 *vs* 98.2 ± 9.5, P<0.001), respectively; respiratory rates before and 15 minutes after treatment starts of the COT group (31.3 ± 2.5 *vs* 27.2 ± 2.3.1, P<0.001) and HFOT group (31.6 ± 2.6 *vs* 28.1 ± 2.2, P<0.001), respectively. Systolic blood pressure and PO_2_ values also improved after treatment starts (P<0.001). We found the Borg’s modified scales significantly improved 60 minutes after treatment starts in both groups, but there was no significant difference. We found significant differences in patient comfort, skill difficulty, requirement, load intensity, and operative time (P<0.001) (**Table 3**).

**Table 2:** Clinical parameters and laboratory tests for 124 patients.

	COT group	HFOT group		
	(n=64)	(n=60)	*t*	P value
Heart rate (beat/min)				
Before treatment	113.2 ± 10.5	114.6 ± 12.1	-0.075	0.94
15 min	113.5 ± 11.8	111.6 ± 10.7	-8.637	<0.001
30 min	105.3 ± 11.1	98.2 ± 9.5	3.705	<0.001
t	-8.718	-6.912		
P value	<0.001	<0.001		
60 min	97.4 ± 8.1	92.1 ± 6.1	1.925	0.043
Respiratory rate (breath/min)				
Before treatment	31.3 ± 2.5	31.6 ± 2.6	-2.93	0.079
15 min	27.2 ± 2.3	24.1 ± 2.2	-1.170	0.247
t	8.614	7.211		
P value	<0.001	<0.001		
30 min	25.4 ± 1.6	23.1 ± 1.8	8.068	<0.001
60 min	25.2 ± 1.3	22.5 ± 1.4	10.115	<0.001
Systolic blood pressure (mmHg)				
Before treatment	137 ± 20	135 ± 22	0.913	0.365
15 min	132 ± 18	134 ± 17	-1.033	0.306
30 min	126 ± 15	124 ± 12	0.088	0.930
t	1.281	1.011		
P value	<0.001	<0.001		
60 min	123 ± 21	121 ± 18	0.0135	0.126
SPO_2_(%)				
Before treatment	93.2 ± 1.8	93.5 ± 1.6	-1.966	0.954
15 min	93.7 ± 2.1	95.3 ± 1.9	-4.655	<0.001
30 min	93.9 ± 2.2	96.4 ± 2.1	-6.916	<0.001
60 min	94.2 ± 1.7	97.8 ± 2.1	-9.993	<0.001
pH value				
Before treatment	7.41 ± 0.12	7.51 ± 0.16	0.102	0.357
60 min	7.36 ± 0.08	7.32 ± 0.12	0.023	0.263
t	1.354	2.36		
P value	0.078	0.012		
PO_2_ (mmHg)				
Before treatment	59.2 ± 3.1	58.9 ± 2.9	-20437	0.18
60 min	62.1 ± 3.4	66.2 ± 3.3	-5.936	<0.001
t	-6.747	-12.403		
P value	<0.001	<0.001		
PCO_2_ (mmHg)				
Before treatment	38.8 ± 5.6	38.2 ± 5.1	-1.077	0.147
60 min	41.6 ± 4.9	40.7 ± 4.6	0.025	0.335
t	1.367	1.205		
P value	0.812	0.253		
HCO_3_				
Before treatment	23.7 ± 2.1	22.9 ± 1.9	-1.87	0.087
60 min	23.2 ± 2.4	23.2 ± 2.8	-2.361	0.721
t	-3.657	-1.257		
P value	0.398	0.127		
Lactic acid (mmHg)				
Before treatment	2.82 ± 2.0	2.79 ± 1.9	0.235	0.105
60 min	2.79 ± 1.9	2.69 ± 1.7	0.388	0.154
t	1.492	2.351		
P value	0.263	0.121		

COT, conventional oxygen therapy; HFOT, high-flow oxygen therapy; SpO_2,_ oxygen saturation; PO_2_, partial pressure of oxygen; oxygen saturation; PCO_2_, pressure of carbon dioxide; HCO_3_, bicarbonate.

**Table 3:** Assessment of activity-related breathlessness.

	COT group	HFOT group		
	(n=64)	(n=60)	t	P value
Borg’s modified scale				
Before treatment	6.81 ± 1.56	6.77 ± 1.71	1.129	0.264
60 min	5.51 ± 1.41	5.49 ± 1.53	0.535	0.594
t	1.763	3.721		
P value	<0.043	<0.001		
Comfort				
60 min	5.72 ± 1.06	7.54 ± 1.53	6.934	<0.001
24-hour ventilation (n)				
Noninvasive	10	4	1	0.5
Invasive	2	2		
7-day mortality (n)	0	0		
NYHA (n)				
III	56	54	1.732	0.182
IV	8	6		
Glasgow Coma Scale	13.01 ± 1.51	12.77 ± 1.43	0.866	0.39
5-point Likert scale				
Skill difficulty	2.34 ± 0.48	2.87 ± 0.71	-4.886	<0.001
Requirement	1.94 ± 0.56	2.2 ± 0.52	-3.540	<0.001
Load intensity	1.85 ± 0.43	2.34 ± 0.51	-6.502	<0.001
Operating risk	1.58 ± 0.36	1.69 ± 0.47	-2.334	0.63
Willingness	1.63 ± 0.41	1.79 ± 0.53	-1.622	0.11
Operative time (min)	0.82 ± 0.21	0.98 ± 0.16	-1.124	<0.001

NYHA, New York Heart Association Functional Classification.

## Discussion

In the treatment of elderly patients with ACPE following hip fractures and surgery, HFOT demonstrates more rapid improvement than COT in heart rate, respiratory rate, SPO_2_, and PO_2_ 15 and 60 minutes after treatment starts. HFOT improves SPO_2_ and PO_2_ more effectively. In addition, HFOT shows greater patient comfort and tolerability than COT. However, HFOT and COT may show equal improvement in respiratory rate, systolic blood pressure, pH, PCO_2_, HCO_3_, and lactic acid. HFOT is easier to implement because it requires less technical skills, training, and nursing workload. HFOT and COT may show equal operating risk and nurses’ willingness.

Globally, 1.7 million hip fractures take place annually. Accidental hospitalization is a common for patients over 65 years, particularly those with hip fractures. Robbins et al. (7) reported that the mortality rate of post-hip fracture was 21% among the patients without prevalent coronary heart disease. Zhao et al. (2) found patients whose age over 75 years old, concomitant coronary heart disease, hemoglobin < 100 g/L are more likely to develop perioperative acute heart failure. ACPE is caused by a failure of the left ventricle to contract forcefully enough. As a result, the left ventricle is unable to pump blood efficiently, which in turn increases the back pressure in the left atrium and lungs (24). This pressure forces the fluid out of the capillaries and into the lungs, causing fluid accumulation in the lungs. Oxygen therapies improve ventilation through the following mechanisms: (1) counterbalancing the mechanical load imposed by residual end-expiratory alveolar pressure in exacerbations; (2) combating atelectasis; and (3) providing a mechanical stent of the upper airways. The therapies initially keep PO_2_ values ≥ 90% (24). COT is commonly performed using nasal prongs, cannulas, or masks (23). However, the maximum oxygen flow rate is only 15 L/minute, which is far lower than an ACPE patient demand (25). Another drawback is the difficulty in meeting the need of gas heating and humidification (26). Therefore, nasal cannulas designed to administer heated and humidified air and oxygen mixtures at high flow rates (> 60 L/minute) become popular. HFOT enhances patient comfort and tolerance. It delivers reliable high levels of FiO_2_, which improves ventilatory efficiency and reduces the work of breathing (24). It provides back pressure to enhance airway patency during expiration, permitting more complete emptying of the air in the lungs. Ko et al. (16) treated 67 patients with acute pulmonary edema combined with heart failure. Among them, 34 patients were treated with HFOT, and 33 patients were treated with COT. Before treatments, the SPO_2_ values of the HFOT and COT groups were 92.83 ± 3.63 and 92.55 ± 4.01, respectively (P=0.765); and the PO_2_ values were 69.84 ± 14.79 and 71.91 ± 19.78, respectively (P=0.629). The SPO_2_ values measured 60 minutes after treatment starts were 97.38 ± 2.51 and 93.39 ± 2.46, respectively (P<0.001). They concluded that HFOT should be the initial oxygen therapy. Those values are comparable to our data. Chang et al. (17) retrospectively reviewed 104 patients with heart failure. The patients were treated with HFOT (n=58) and COT (n=46). The two therapies were equal in preventing extubating failure and reintubation. However, the assessments were performed 72 hours after extubation and were not based on SPO_2_ and PO_2_. Şener et al. (18) treated 112 patients with hypertensive pulmonary edema. Among them, 50 patients were treated with COT, and 62 patients were treated with HFOT. Before treatments, the mean SPO_2_ values of the HFOT and COT groups were 81.67 ± 5.6 and 86.04 ± 6.43, respectively (P <0.001); and the mean PO_2_ values were 58.19 ± 6.05 and 63.54 ± 9.28, respectively (P<0.001). One hour after treatment starts, the mean SPO_2_ values were 163.62 ± 75.84 and 80.24 ± 21.86, respectively (P<0.001). Those values are also comparable to our data. HFOT is much more effective as it shortens the length of stay in an emergency or intensive care unit. HFOT also provides better results in terms of blood gas analysis, heart rate, and respiratory rate.

In this study, both HFOT and COT treatments increase SPO_2_ values and arterial oxygen partial pressure, reducing heart rate, respiratory rate, and systolic blood pressure. However, HFOT produces earlier and more significant effects due to specified oxygen inhalation and positive pressure in the airway. The improvement of hypoxia further reduces the respiratory rate and heart rate, reducing oxygen consumption and cardiopulmonary burden.

In addition to PO_2_ and SPO_2_, some other indicators are used to evaluate ACPE. Chest X-rays are commonly used, but the characteristic features have only moderate specificity (range, 75%–83%) and poor sensitivity (range, 50%–68%) (27). Transthoracic pulmonary ultrasound can be used to evaluate ACPE, but the drawbacks are technique dependence and limited specificity in the identification of pulmonary edema (28).

In the management of ACPE following hip fractures and surgery, HFOT is selected based on the severity of hypoxaemia, underlying mechanisms, and patient’s breathing pattern and exercise tolerance (29). According to our experience, the best duration of HFOT may be 1–2 hours. Indications for HFOT are cardiogenic pulmonary edema, secretion retention, and hypoxemic respiratory failure. Contraindications include poor cooperation, severe nasal obstruction, copious nose bleeding, recent nasal trauma, surgery representing potential, and mild symptoms without the need for HFOT. HFOT provides lower positive pressure and positive end-expiratory pressure, reducing the severity of hypoxemia. In this study, however, the PCO_2_ values did not decrease because the parameters were within the normal range (35–45 mmHg) by adjusting breathing.

The advantages of HFOT include (1) physiological benefits in patient comfort and outcomes in various clinical settings; (2) efficiency in hypoxemic acute respiratory failure; (3) easy implementation and management; (4) a minimal risk of developing skin breakdown; (5) low nurse workload; (6) stability of the nasal cannula; (7) no claustrophobia; and (8) eating, drinking, and communicating permitted. The disadvantages are runny nose, pneumothorax in newborns such as air-leak syndrome, feeling hot, noise, limited movement, and risk of delayed intubation. Infrequent problems are nasal mucosal irritation, discomfort, smelling alteration, and dislocation of the nasal cannula. When COT cannot achieve clinical needs (e.g. SPO_2_ < 90% due to intolerance of maximum inspired oxygen concentration or certain oxygen inhalation), converting COT to HFOT is not appropriate for patients with nasal injuries or diseases.

This study has limitations. First, we exclude the patients with SPO_2_ values <90% after 10 minutes of treatment starts and patients with worsening hypoxia, which may produce selection bias. Second, therapeutic effects are difficult to generalize in the development of ACPE in first-diagnosed and severe cases. Third, concomitant diseases may affect the assessments. Further prospective multicenter trials and a period of follow-up are required to validate the usefulness of HFOT in ACPE following hip fractures and surgery.

## Conclusion

In the treatment of elderly patients with ACPE following hip fractures and surgery, HFOT may be performed to improve ventilation when acute cardiogenic pulmonary edema does not significantly improve within 15 minutes of COT.

## Data Availability

The raw data supporting the conclusions of this article will be made available by the authors, without undue reservation.
